# Randomized trial of weight loss on circulating ghrelin levels among breast cancer survivors

**DOI:** 10.1038/s41523-021-00260-6

**Published:** 2021-05-11

**Authors:** Leah Puklin, Brenda Cartmel, Maura Harrigan, Lingeng Lu, Fang-yong Li, Tara Sanft, Melinda L. Irwin

**Affiliations:** 1 Yale University School of Public Health, New Haven, CT USA; 2 Yale Cancer Center, Yale University School of Medicine, New Haven, CT USA

**Keywords:** Randomized controlled trials, Cancer epidemiology, Breast cancer, Breast cancer, Risk factors

## Abstract

Obesity among breast cancer survivors is associated with increased risk for recurrence and mortality. The hormone ghrelin plays a role in initiating appetite and thus regulating body weight. This study aims to determine the effect of a lifestyle intervention on ghrelin levels in breast cancer survivors with a body mass index (BMI) ≥ 25 kg/m^2^. The Lifestyle, Exercise, and Nutrition (LEAN) study was a 6-month randomized trial, examining the effectiveness of a weight loss intervention versus usual care in 151 breast cancer survivors with BMI ≥ 25 kg/m^2^. Ghrelin was measured in fasting baseline and 6-month blood samples. Baseline associations between ghrelin, body composition, and blood biomarkers were examined. Six-month change in ghrelin was compared between study arms. Ghrelin measurements were available for 149 women. At baseline, ghrelin was correlated with age (*r* = 0.28, *p* < 0.001) and inversely correlated with weight (*r* = −0.18, *p* = 0.03), lean body mass (*r* = −0.18, *p* = 0.02), and leptin (*r* = −0.18, *p* = 0.03). Over 6 months, ghrelin increased by 144 pg/mL (7.2%) in the intervention and decreased by 466 pg/mL (32.5%) in the usual care (*p* = 0.07). Among all women, greater weight loss was associated with an increase in ghrelin (*p* = 0.01). These findings indicate that weight loss, achieved through a lifestyle intervention, is associated with higher ghrelin levels in breast cancer survivors which may be informative for developing sustainable weight loss programming for this population. Future research should investigate the long term impacts of lifestyle interventions on ghrelin levels in the context of weight maintenance and weight regain.

## Introduction

In 2019, the American Cancer Society estimated that there were approximately 3,861,520 breast cancer survivors living in the United States with this number expected to increase to 4,957,960 by 2030^1^. The increase in female breast cancer survival rates is partially attributable to widespread mammography use and improvements in treatments^1^. As the population of breast cancer survivors grows, it becomes increasingly important to understand the specific needs associated with cancer survivorship.

Weight gain among women with breast cancer is a common problem. Between 50% and 96% of women experience significant weight gain during treatment ranging from 2.5 to 6.2 kg^2,3^. Factors related to post-diagnosis weight gain include chemotherapy, postmenopausal status, decreased physical activity, and increased total caloric intake^4–6^. Obesity and post-treatment gain in adipose tissue places breast cancer survivors at an elevated risk for recurrence and breast cancer-specific mortality for decades^7^. Research has shown that every 5 kg increase in weight is associated with a 13% increase in breast cancer-specific mortality^8,9^.

Ghrelin, referred to as the “hunger hormone”, is a 28-amino acid peptide hormone that plays a major role in regulating appetite^10^. Ghrelin was first isolated in 1999 in rat gastric mucosa and since then it has been identified that over 90% of ghrelin in the human body is produced in the stomach and duodenum^11–14^. Ghrelin is primarily produced in the gastric fundus by endocrine cells and stimulates pituitary Growth Hormone (GH) secretion through the GH secretagogue receptor^13^. Ghrelin binds to hypothalamic receptors to initiate signaling leading to an increase in appetite and food intake^13,15^. Levels of ghrelin fluctuate naturally throughout the day, with higher levels before meals (preprandial) and during the night compared to lower levels following mealtime (postprandial)^16^. Multiple studies have found that plasma ghrelin levels are downregulated in patients with obesity, meaning individuals with a body mass index (BMI) ≥ 25 kg/m^2^ have lower levels of circulating ghrelin compared to those with a BMI < 25 kg/m^2^ ^10,16,17^. It has been well documented that there is a linear inverse correlation between circulating ghrelin levels and BMI^10,16,17^. The mechanisms for this relationship remain unclear, however, it is hypothesized that individuals with obesity may experience a dysfunction in the gene for ghrelin that disturbs the normal production and actions of the hormone^10^.

Unlike other appetite regulating hormones, ghrelin has been shown to play a role in long term body weight regulation^13^. Administering ghrelin to animals caused an increase in food consumption and a decrease in energy expenditure, which led to weight gain^11,18^. Comparatively, blocking ghrelin signaling was shown to decrease food intake and result in a decrease in overall body weight^11,18^. These findings suggest ghrelin could participate in a negative feedback loop that regulates body weight. The response of circulating ghrelin to weight loss has been examined primarily in the setting of surgical weight loss interventions and to a lesser extent in lifestyle interventions^10,15–17,19,20^.

The current literature reports inconsistent findings regarding the mechanisms by which bariatric surgery induced weight loss effects ghrelin concentrations. As reported in a 2011 review, most surgical weight loss procedures resulted in significant weight loss, however, the postoperative serum ghrelin levels differed among the various surgical procedures and length of follow-up^21^. The authors argued that only surgical procedures which completely removed the gastric fundus, such as sleeve gastrectomy, achieved both significant changes in BMI and decreases in ghrelin levels likely because fewer ghrelin-producing cells exist^16^. Cummings et al. studied individuals who underwent Roux-en-Y gastric bypass surgery (RYGB) and showed that serum ghrelin levels no longer fluctuated in relation to meals and were lower than those in both the normal weight controls (BMI = 27.4 kg/m^2^) and matched obese controls (BMI = 40.0 kg/m^2^), regardless of the amount of weight lost^19^. Comparatively, a recent meta-analysis of sixteen studies looked at the differences between short term (≤3 months) and long term (>3 months) effects of RYGB surgery on ghrelin and weight loss^22^. This paper reported ghrelin levels after RYGB surgery were significantly lower than pre-surgery levels in the short term, however, ghrelin levels were markedly higher in the long term^22^. Further investigation is warranted to fully understand the role ghrelin plays on long term weight loss and maintenance after bariatric surgery.

Exploring the role circulating ghrelin plays in lifestyle weight loss interventions in breast cancer survivors is challenging given the lack of randomized controlled trials^9^. However, two randomized behavioral intervention trials which enrolled women without cancer have reported on the effect of the interventions on serum ghrelin levels. A prospective randomized controlled trial that enrolled 173 postmenopausal women with a BMI ≥ 25 kg/m^2^, found circulating ghrelin levels significantly increased during the 12-month exercise intervention (45-min moderate aerobic exercise 5 days/week) (+32 ± 16 pg/ml; *p* < 0.05 compared to baseline) while the usual care (stretching) showed a non-significant increase in ghrelin over the same time period^23^. A more recent randomized-controlled trial by Mason et al. examined the independent and combined effects of a 12-month dietary weight loss and/or aerobic exercise intervention on total ghrelin levels in 398 post-menopausal women with a BMI ≥ 25 kg/m^2^ ^20^. Compared to the usual care group, ghrelin increased significantly in the combined diet and exercise group (+100 pg/mL [7.4%], *p* = 0.008) but not in either the diet only (+87 pg/mL [6.5%], *p* = 0.07) or exercise only (+14 pg/mL [1.0%], *p* = 0.53) groups over the 12-month study period^20^. Both studies noted that the magnitude of change in circulating ghrelin was associated with amount of weight loss.

Little is known about circulating ghrelin levels among breast cancer patients compared to women without breast cancer. To date, among breast cancer patients, the focus has been on the impact of therapies such as chemotherapy on ghrelin levels^24^. Studies conducted in ovarian and prostate cancer patients have found that ghrelin concentrations did not differ between patients with tumors compared to controls^25,26^. Current studies have also presented conflicting results regarding the pro-proliferative effects or inhibitory effects of ghrelin on breast cancer^24^.

Given the risks of reduced disease-free survival and overall survival associated with obesity and weight gain among breast cancer survivors, it is important to understand the relationship between lifestyle interventions which result in weight loss and serum ghrelin levels among this population^8,9,27^. The Lifestyle, Exercise and Nutrition (LEAN) study examined the effects of a behavioral, dietary, and physical activity intervention through in-person and telephone counseling sessions, among 100 breast cancer survivors (with an additional 51 patients in the second iteration of the study) with a BMI ≥ 25 kg/m^2^. This initial three-armed randomized trial found an average 6.4% (in-person counseling) and 5.4% (telephone counseling) reduction in body weight for women in the lifestyle intervention arms compared to a 2.0% decrease in the usual care group (*p* < 0.05 for in-person and telephone counseling vs. usual care)^28^. The purpose of our analysis was to examine the effect of the LEAN weight loss intervention versus usual care on ghrelin levels and other biomarkers which indicate inflammation (C-reactive protein) and regulate energy balance, hunger, and satiety (insulin, adiponectin, leptin), among an enlarged group of breast cancer survivors with a BMI ≥ 25 kg/m^2^ ^29,30^.

Understanding the relationship between circulating ghrelin and weight change induced by a lifestyle intervention in overweight or obese breast cancer survivors may be informative for developing and adapting existing sustainable weight loss programming for this population. We hypothesized that women randomized to the weight loss intervention would have an increase in serum ghrelin levels over the 6-month study compared to the usual care group and additionally, that the magnitude of increase in serum ghrelin would be proportional to the amount of weight lost.

## Results

### Study population and recruitment

#### Baseline characteristics

**Fig. 1:**
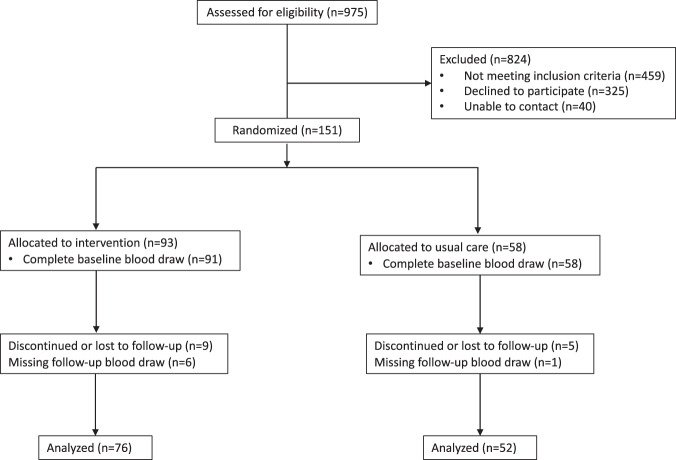
Consort diagram. Flow diagram of individuals enrolled in the lifestyle, exercise and nutrition (LEAN) trial.

Full recruitment details are illustrated in Fig. 1 and baseline characteristics are reported in Table 1. Of the 975 women assessed for eligibility, 151 women were randomized, 149 had baseline blood samples (intervention = 91, usual care =58), and 128 had 6-month blood samples (intervention = 76, usual care = 52). Mean age of participants at baseline was 58.0 ± 7.8 years (mean ± SD, unless otherwise noted) and women were on average 2.9 ± 2.5 years out from diagnosis at the time of enrollment in LEAN. Women were predominately post-menopausal (83%), non-Hispanic white (89%), and highly educated, with 61% holding at least a college degree. Most women had been diagnosed with Stage I or II breast cancer (50% and 24% respectively), with 16% diagnosed as Stage 0 (ductal carcinoma in situ: DCIS). A majority of women had received adjuvant treatment from chemotherapy and/or radiation (88%) and reported some form of previous or current endocrine therapy with tamoxifen and/or aromatase inhibitors (64%).

**Table 1:** LEAN 1 and LEAN 2 study participant characteristics.

Characteristic	Total (*n* = 149)	Intervention (*n* = 91)	Usual Care (*n* = 58)	*P* value^a^
Age, years, mean (SD), range (*n* = 149)	58.0 ± 7.8 32-73	59.0 ± 7.3 44-73	56.3 ± 8.4 32-72	0.04
Postmenopausal, *n* (%) (*n* = 149)	124 (83)	77 (85)	47 (81)	0.57
Race/Ethnicity, *n* (%) (*n* = 149)				0.53
White (non-Hispanic)	131 (88)	82 (90)	49 (85)	
Black or African American	10 (7)	5 (6)	5 (9)	
Hispanic	6 (4)	3 (3)	3 (5)	
Other	1 (1)	0 (0)	1 (2)	
Declined to report	1 (1)	1 (1)	0 (0)	
Education, *n* (%) (*n* = 149)				0.17
High school degree	19 (13)	10 (11)	9 (16)	
Some college degree	39 (26)	22 (24)	17 (29)	
College degree	38 (26)	29 (32)	9 (16)	
Graduate degree	53 (36)	30 (33)	23 (40)	
Time from diagnosis to LEAN enrollment, years, mean (SD) (*n* = 147)	2.9 ± 2.5	2.7 ± 2.0	3.2 ± 3.1	0.25
Body weight, kg, mean (SD) (*n* = 149)	87.8 ± 17.7	85.0 ± 16.9	92.3 ± 18.1	0.01
Percent body fat (SD) (*n* = 149)	43.2 ± 4.9	43.3 ± 4.5	42.9 ± 5.5	0.63
Baseline BMI, kg/m^2^, mean (SD) (*n* = 149)	33.2 ± 6.4	32.2 ± 6.0	34.6 ± 6.7	0.03
BMI (kg/m^2^) (*n* = 149)				0.02
Overweight BMI < 30	61 (41)	44 (48)	17 (29)	
Obese BMI ≥ 30	88 (59)	47 (52)	41 (71)	
Disease stage, *n* (%) (*n* = 149)				0.81
DCIS (stage 0)	25 (17)	13 (14)	12 (21)	
Stage I	74 (50)	46 (51)	28 (48)	
Stage II	36 (24)	22 (24)	14 (24)	
Stage III	11 (7)	8 (9)	3 (5)	
Unknown	3 (2)	2 (2)	1 (2)	
Adjuvant treatment after surgery, *n* (%) (*n* = 149)				0.38
None	17 (11)	8 (9)	9 (16)	
Radiation only	57 (38)	34 (37)	23 (40)	
Chemotherapy only	23 (15)	17 (19)	6 (10)	
Radiation and chemotherapy	52 (35)	32 (35)	20 (35)	
Current endocrine therapy, *n* (%) (*n* = 128)				0.07
Aromatase inhibitors (AI’s) only	30 (23)	21 (26)	9 (19)	
Tamoxifen	43 (34)	20 (25)	23 (48)	
Both	9 (7)	6 (8)	3 (6)	
None	46 (36)	33 (41)	13 (27)	

^a^*T*-tests for continuous variables and chi-squared tests or Fischer’s exact tests for categorical variables.

At baseline, age, body weight, BMI, and BMI category were found to be statistically significantly different between the intervention versus usual care groups, (*p* ≤ 0.05). Individuals randomized to the intervention were on average 59.0 ± 7.3 years old and significantly older than those in the usual care group (56.3 ± 8.4 years old) (*p* = 0.04). Those randomized to intervention were found to have a baseline body weight of 85.0 ± 16.9 kg whereas baseline body weight in the usual care group was 92.3 ± 18.1 kg (*p* = 0.01). Baseline BMI was significantly lower in the intervention group (32.2 ± 6.0 kg/m^2^) compared to usual care (34.6 ± 6.7 kg/m^2^) (*p* = 0.03). There were no other statistically significant differences in baseline characteristics between randomization groups.

#### Adherence to intervention

Sixty percent of participants randomized to intervention attended all 11 weight loss counseling sessions and 80% attended at least 8 of the counseling sessions.

#### Baseline associations

Baseline associations between circulating ghrelin, measures of body composition, and serum biomarkers are described in Table 2. At baseline, there was a significant positive correlation between circulating ghrelin and age (*r* = 0.28, *p* < 0.001). Circulating ghrelin levels at baseline was significantly inversely correlated with weight (*r* = −0.18, *p* = 0.03), lean body mass (*r* = −0.18, *p* = 0.02), and leptin (*r* = −0.18, *p* = 0.03). Baseline ghrelin was not significantly associated with BMI, total body fat, insulin, adiponectin, or C-reactive protein.

**Table 2:** Unadjusted baseline Pearson correlation coefficients of ghrelin levels with age, measurements of body composition, and serum biomarkers in all study participants (*n* = 149).

	Correlation	*P* value
Age	0.28	0.001
Weight (kg)	−0.18	0.03
BMI (kg/m^2^)	−0.14	0.08
Total Body fat (kg)	−0.13	0.11
Lean Body Mass (kg)	-0.18	0.02
Leptin (ng/mL)	−0.18	0.03
Insulin (µU/mL)	−0.13	0.11
Adiponectin (µg/mg)	0.05	0.54
C-Reactive Protein (mg/L)	−0.04	0.60

#### Changes in body weight

Adjusting for age and baseline weight, women randomized to the intervention arm lost an average of 5.0 kg (5.8%) from baseline to 6 months (*p* < 0.0001) (Table 3). Women randomized to the control arm did not have a statistically significant weight loss at 6 months (baseline vs. 6-month difference= 0.3 kg, 0.3%, *p* = 0.66). The change in weight among women in the intervention arm compared to the control arm over the 6-month intervention was statistically significant (*p* < 0.0001).

**Table 3:** Baseline, 6-month, and change in ghrelin levels and weight by randomization group.

Outcomes	Month	Intervention group, mean (95% CI)	Control group, mean (95% CI)	Group difference, mean (95% CI)	*P* value^c^
Ghrelin (pg/mL)	Baseline	1989 (1458, 2521), *n* = 91	1434 (994, 1873), *n* = 58	556 (−129, 1240)	0.11
	6-month	2043 (148, 2606), *n* = 76	1067 (692, 1442), *n* = 52	976 (305, 1646)	0.01
	6-month change	144 (−322, 610)^a^, *n* = 76	−466 (−1019, 88)^a^, *n* = 52	610 (−61, 1281)^a^	0.07
	% change	7.2%	-32.5%		
	*P* value^d^	0.54	0.09		
Weight (kg)	Baseline	85.0 (81.5, 88.5), *n* = 91	92.3 (87.5, 97.5), *n* = 58	−7.3 (−13.1, -1.5)	0.01
	6-month	79.6 (75.5, 83.6), *n* = 77	90.6 (85.4, 95.8), *n* = 53	−11.0 (−17.5, -4.6)	0.001
	6-month change	−5.0 (−5.9, -4.1)^b^, *n* = 77	−0.3 (−1.4, −0.9)^b^, *n* = 53	−4.7 (−6.1, -3.3)^b^	<0.0001
	% change	−5.8%	−0.3%		
	*P* value^d^	<0.0001	0.66		

^a^Adjusted for baseline ghrelin, age, and baseline BMI.

^b^Adjusted for age, baseline weight.

^c^*T-*test.

^d^Paired *T-*test.

#### Changes in serum ghrelin

At baseline, ghrelin levels appeared to be lower in the usual care (1434 pg/mL (95% CI: 994, 1873 pg/mL)) compared to that in the intervention group (1989 pg/mL (95% CI: 1458, 2521 pg/mL)) but the difference was not statistically significant (*p* = 0.11) (Table 3). After the 6-month intervention ghrelin levels differed significantly between study arms (usual care = 1067 pg/mL (95% CI: 692, 1442 pg/mL), intervention = 2043 pg/mL (95% CI: 148, 2606 pg/mL), *p* = 0.01). Adjusting for baseline ghrelin, baseline BMI, and age, the change in serum ghrelin from baseline to 6 months between the usual care (−466 pg/mL (95% CI: (−1019, 88 pg/mL)) and intervention arms (144 pg/mL (95% CI: −322, 610 pg/mL)) were approaching a statistically significant difference (*p* = 0.07). Serum ghrelin levels decreased by 32.5% among the usual care group and increased by 7.2% among the intervention group over 6 months. A sensitivity analysis was performed excluding women who had not fasted for a minimum of 8 h prior to the blood draw (*n* = 7), however, the results did not change significantly (results not shown).

#### Relationship between changes in ghrelin levels and weight

We found that weight change is a significant predictor of changes in ghrelin when controlling for baseline BMI, baseline ghrelin, and age (*p* = 0.01). Specifically, for every 1 kg loss in body weight, there was a 93.4 pg/mL (Standard Error = 36.8) increase in ghrelin levels among all study participants (*n* = 128).

## Discussion

Studies examining the role of circulating ghrelin levels on weight loss and weight maintenance have primarily been conducted in the setting of surgical interventions and less so in lifestyle weight loss interventions^10,15–17,19,20^. Obesity, weight loss, and breast cancer are interrelated and we sought to understand the association between an individualized weight loss intervention and circulating ghrelin levels among overweight or obese breast cancer survivors.

The 6-month LEAN intervention led to significant weight loss in the intervention group compared to those in the usual care group. While the change in ghrelin levels between groups was not statistically significant, we did observe a 7.2% increase in ghrelin levels (+144 pg/mL) in the intervention group. We also found a non-significant 32.5% decrease in ghrelin levels (-466 pg/mL) in the control group over the 6-month intervention which we had not expected. As average weight was stable over time for the women in the control arm, we do not know the reason for the observed decrease in ghrelin in this group. However, we hypothesize that it may be due, in part, to differences in diet composition and/or changes in body composition (i.e. body fat) over time and this association should be investigated further in future studies. The magnitude of increase in ghrelin levels among the intervention arm was consistent with two randomized-controlled intervention trials in women without breast cancer^20,23^. Mason et al. observed a 7.4% increase in circulating ghrelin levels among the exercise and diet arm compared to a non-significant 6.5% increase among the diet only arm and a 1.0% increase among the exercise only arm^20^. Similarly, Foster-Schubert et al. showed women randomized to the exercise arm had a significant increase in ghrelin levels over the 12-month intervention (32 ± 16 pg/ml, *p* < 0.05)^23^.

We found inverse associations between ghrelin and body weight, BMI, leptin, and lean body mass. Our findings are consistent with those of Foster-Schubert et al. who reported inverse associations with body weight (*r* = −0.29, *p* < 0.0001), BMI (*r* = −0.29, *p* < 0.0001), LBM (*r* = −0.24, *p* = 0.001), and leptin (*r* = −0.14, *p* = 0.08) and Tschop et al. who reported similar inverse association with BMI (*r* = −0.5, *p* < 0.01) and leptin (*r* = −0.39, *p* < 0.05)^17,23^. Our results, along with previous studies, suggest that ghrelin levels are downregulated in individuals with a BMI ≥ 25 kg/m^2^.

Our study found a significant relationship between increasing ghrelin levels with decreasing weight. Specifically, per 1 kg decrease in weight, ghrelin levels increased by 93.4 pg/mL. Similarly, Mason et al. found a decrease in ghrelin levels among those who lost no weight and an incremental increase in ghrelin levels among those who lost <5%, 5–10%, and >10% of their body weight over the study period^20^. Foster-Schubert et al. found ghrelin levels increased commensurately with the amount of weight lost over the 12-month intervention^23^. These results indicate ghrelin plays a role in the adaptive responses to weight loss.

The mechanisms by which weight loss leads to an increase in circulating ghrelin is not fully understood nor which aspects of body composition regulate ghrelin^23^. To our knowledge, no study has prospectively examined changes in weight and ghrelin long term. This magnifies the need to understand the long term impacts of physical activity and diet-induced weight loss on ghrelin levels as well as the mechanisms by which body composition impacts ghrelin levels in the body.

A potential limitation of our findings is the intervention was limited to 6 months, and therefore, long term effects were not captured. Further longitudinal research and long term follow-up assessments of weight and ghrelin is warranted. Results of this study should be viewed in the context that participants were predominately non-Hispanic white and highly educated which may limit the generalizability of our findings. Also, our sample size was limited when assessing trends in change in ghrelin with amount of weight change. Only a few randomized weight loss trials in breast cancer survivors have been published, however, and the majority have had smaller sample sizes. Given the results presented above were performed as a secondary analysis of the LEAN intervention, we were unable to gather additional data on changes in self-reported satiety and hunger among the participants. Therefore, we cannot comment on whether the observed increase in ghrelin levels produce subsequent changes in hunger and satiety that may lead to future weight regain. Additionally, due to a lack of power, we were not able to explore whether the effect is similar among those taking versus not taking endocrine therapy which may be an important effect modifier.

Strengths of this study include a low attrition rate and high adherence to the LEAN intervention. Previously published LEAN results showed significantly greater weight loss in women who completed all counseling sessions compared to those who missed sessions^28^. Another strength of this study is that the majority of women had fasted for a minimum of 8 h prior to the blood draw (97%). Overnight fasting correlates well with 24-hour profiles of ghrelin^31^.

Taken together, the observed increases in circulating ghrelin levels as a response to the LEAN intervention may have the potential to help prevent weight regain in the long term. Therefore, the results presented from this study are a vital contribution to understanding the relationship between lifestyle interventions and ghrelin levels among a population at high risk for cancer recurrence or mortality. With over 65% of breast cancer survivors overweight or obese, identifying the proper programming, timing, and length of behavioral lifestyle interventions to combat the biological adaptations that may influence long term weight regain, may ultimately improve the health outcomes of this growing cancer survivorship population^32^.

In summary, we show that ghrelin levels increased in breast cancer survivors undergoing a 6-month diet and physical activity weight loss intervention. This finding is consistent with previous studies examining populations of women without cancer and supports the notion that future research should be performed to determine the long term effect of changes in ghrelin on weight maintenance, and in turn, its impact on cancer risk and mortality.

## Methods

### Trial design

The Lifestyle Exercise and Nutrition (LEAN) study was a Phase III randomized controlled weight loss trial (NCT02109068 and NCT02110641), registered in January 2011 and November 2013, respectively, evaluating the effectiveness of in-person or telephone-based weight loss counseling versus usual care on changes in body composition, physical activity, diet, and serum biomarkers over 6 months in 100 breast cancer survivors. The detailed protocol and primary results for the LEAN study have been published previously^28^. Based on the initial results, 51 additional participants were recruited and randomized to intervention or usual care to increase the sample size for this study (total *n* = 151). Written informed consent was obtained from all participants in accordance with the protocol approved by the Yale School of Medicine Human Investigation Committee.

### Participants and recruitment

Eligible participants included breast cancer survivors with a BMI ≥ 25 kg/m^2^, diagnosed with stage 0–III breast cancer who had completed chemotherapy and/or radiation therapy at least 3 months before enrollment. Women had to be capable of walking, agree to be randomly assigned, provide informed consent to participate, communicate in English and be accessible by telephone. Women were excluded from the study if they were pregnant or intending to become pregnant in the next year, had experienced (past 6 months) stroke or myocardial infarction, or had severe uncontrolled mental illness. Breast cancer survivors were recruited between June 1, 2011 and February 1, 2016. Participants were identified through medical oncology clinics or self-referred via study brochures in the Breast Center at Smilow Cancer Hospital at Yale-New Haven Hospital and the Yale Cancer Center Survivorship Clinic. Details surrounding the eligibility criteria, recruitment and study design have been described in previously published literature^28,33^.

### Outcome measures

#### Demographic and medical history

Medical record review and questionnaires were used to determine disease stage, surgery, adjuvant therapy, endocrine therapy, self-reported weight, and comorbidities at baseline and 6 months.

#### Body composition measures

Height and weight were measured at baseline and 6 months. All measurements were made by the same staff members and were performed and recorded twice in succession. The mean value was used in the analyses. Dual-energy x-ray absorptiometry scans were performed to assess body fat, and lean body mass (LBM) at baseline and 6 months with a Hologic 4500 scanner. All scans were evaluated by a Radiologic Technician Certified in Bone Density who was blinded to randomization group.

#### Ghrelin and other biomarker analysis

A fasting (≥8 h) blood draw was performed at baseline and 6 months. All serum samples were stored at -80 degree Celsius until assayed. Total serum ghrelin levels were measured using a commercial human ghrelin ELISA (enzyme-linked immunosorbent assays) kit (BMS2192, ThermoFisher Scientific, Waltham, MA). The serum samples from each individual were analyzed in duplicate, and the absorbance was measured at the wavelength of 450 nm with the reference wavelength of 620 nm for correction using a 96-well BioTek Synergy HT microplate spectrophotometer (BioTek, Winooski, VT). The coefficients of variation for human ghrelin ELISA intra-assay was 1.69% in this study. Description of other serum biomarker analyses have been previously described^28^. Serum concentrations of insulin, leptin, and adiponectin were measured using radioimmunoassay kits; and C-reactive protein (CRP) was measured using an automated chemistry analyzer. Baseline and 6-month specimens were assayed simultaneously at the end of the study, and participants from the intervention and the usual care arms were included in each batch of assays. Laboratory technicians were blinded to intervention assignment.

#### Weight loss intervention

The lifestyle intervention for the weight loss group was designed using a combination of behavioral therapy, reducing caloric intake, and increasing physical activity. The program was modified from the Diabetes Prevention Program, updated with 2010 U.S. Dietary Guidelines and adapted to the breast cancer survivor population using the American Institute for Cancer Research/World Cancer Research Fund and American Cancer Society nutrition and physical activity guidelines^34,35^. All counseling sessions provided to the participants were conducted by a Registered Dietitian who is a certified Specialist in Oncology Nutrition, trained in exercise physiology and behavior modification counseling.

The 6-month weight loss intervention involved participants receiving individual counseling sessions once a week for the first month, every two weeks for months two and three, followed by once a month for months four, five, and six. The 11 sessions, each 30 min in duration, provided individualized information on nutrition, exercise, and social-cognitive theory-based behavior strategies.

The dietary counseling instructed participants to reduce energy intake to a range of 1200 to 2000 kcal/day based upon baseline weight and to incur an energy deficiency of 500 kcal/day. This reduction was promoted by maintaining a predominantly plant-based diet with education on portion size, tracking fat grams, reducing simple sugars, increasing fiber, and incorporating mindful eating techniques. The physical activity program was home based, with the goal of 150 min per week of moderate-intensity activity, such as brisk walking. Each participant was provided a pedometer and was coached to increase their daily step count to 10,000 steps per day in addition to reducing sedentary behaviors.

#### Usual care

Study participants assigned to the usual care group were provided the American Institute for Cancer Research nutrition and physical activity brochures and were referred to the Yale Cancer Center Survivorship Clinic, which offers a two-session weight management program.

### Statistical analysis

Baseline characteristics were summarized and compared between randomization arms using t-tests for continuous variables and chi-squared tests or Fischer’s exact test for categorical variables. Of the 151 LEAN participants, 149 had baseline serum ghrelin measurements (91 intervention group, 58 control group). Six-month data were available for 128 women with 14 participants discontinued or lost to follow-up and an additional 7 participants missing follow-up blood draws (76 intervention group, 52 control group) (Fig. 1).

Pearson correlation coefficients were used to examine baseline associations. The mean baseline to 6-month changes were compared between groups using a mixed model repeated measures analysis in an intention to treat (ITT) fashion. This analytical approach uses a maximum-likelihood estimator to handle incomplete data with an assumption of missing at random. A sensitivity analysis was performed excluding those with a fasting status of <8 h (*n* = 7).

Post hoc analysis examined whether changes in ghrelin levels were associated with changing weight in the full study cohort. We ran a multiple linear regression model using change in ghrelin and change in weight as continuous variables. Potential confounders were added as covariates to the models for exploratory analysis including age, baseline ghrelin, and baseline BMI.

All analyses were performed using SAS software version 9.4 (Cary, NC). A two-sided type I error rate of 0.05 was used throughout the data analysis.

### Reporting summary

Further information on research design is available in the Nature Research Reporting Summary linked to this article.

## Supplementary information

Reporting Summary

## Data Availability

The data generated and analyzed during this study are described in the following data record: 10.6084/m9.figshare.14356184^36^ The data are contained in the following files: ‘Consort_Diagram.xlsx’, ‘FinalCode_Table1_2_Ghrelin.sas7bdat’ and ‘FinalCode_Table3_Ghrelin.sas7bdat’. However, these data are not publicly available in order to protect patient privacy. A complete de-identified patient-level dataset, study protocol and statistical analysis plan will be made available to researchers upon request until December 2025 by contacting the corresponding author.

## Code availability

The codes developed during this study are available upon reasonable request. Analyses were performed using SAS software version 9.4 (Cary, NC).

## References

[1] Cancer Treatment & Survivorship: Facts & Figures 2019–2021, https://www.cancer.org/content/dam/cancer-org/research/cancer-facts-and-statistics/cancer-treatment-and-survivorship-facts-and-figures/cancer-treatment-and-survivorship-facts-and-figures-2019-2021 (2019–2021).

[2] Irwin ML (2005). Changes in body fat and weight after a breast cancer diagnosis: influence of demographic, prognostic, and lifestyle factors. J. Clin. Oncol..

[3] Vance V, Mourtzakis M, McCargar L, Hanning R (2011). Weight gain in breast cancer survivors: prevalence, pattern and health consequences. Obes. Rev..

[4] Rock CL (1999). Factors associated with weight gain in women after diagnosis of breast cancer. Women’s Healthy Eating and Living Study Group. J. Am. Diet. Assoc..

[5] Demark-Wahnefried W (2001). Changes in weight, body composition, and factors influencing energy balance among premenopausal breast cancer patients receiving adjuvant chemotherapy. J. Clin. Oncol..

[6] Demark-Wahnefried W, Winer EP, Rimer BK (1993). Why women gain weight with adjuvant chemotherapy for breast cancer. J. Clin. Oncol..

[7] Jiralerspong S, Goodwin PJ (2016). Obesity and breast cancer prognosis: evidence, challenges, and opportunities. J. Clin. Oncol..

[8] Kroenke CH, Chen WY, Rosner B, Holmes MD (2005). Weight, weight gain, and survival after breast cancer diagnosis. J. Clin. Oncol..

[9] Nichols HB (2009). Body mass index before and after breast cancer diagnosis: associations with all-cause, breast cancer, and cardiovascular disease mortality. Cancer Epidemiol. Biomark. Prev..

[10] Makris MC (2017). Ghrelin and obesity: identifying gaps and dispelling myths. A reappraisal. Vivo.

[11] Tschöp M, Smiley DL, Heiman ML (2000). Ghrelin induces adiposity in rodents. Nature.

[12] Kojima M (1999). Ghrelin is a growth-hormone-releasing acylated peptide from stomach. Nature.

[13] Klok MD, Jakobsdottir S, Drent ML (2007). The role of leptin and ghrelin in the regulation of food intake and body weight in humans: a review. Obes. Rev..

[14] Ariyasu H (2001). Stomach is a major source of circulating ghrelin, and feeding state determines plasma ghrelin-like immunoreactivity levels in humans. J. Clin. Endocrinol. Metab..

[15] Hansen TK (2002). Weight loss increases circulating levels of ghrelin in human obesity. Clin. Endocrinol..

[16] Kotidis EV (2006). Serum ghrelin, leptin and adiponectin levels before and after weight loss: comparison of three methods of treatment–a prospective study. Obes. Surg..

[17] Tschöp M (2001). Circulating ghrelin levels are decreased in human obesity. Diabetes.

[18] Nakazato M (2001). A role for ghrelin in the central regulation of feeding. Nature.

[19] Cummings DE (2002). Plasma ghrelin levels after diet-induced weight loss or gastric bypass surgery. N. Engl. J. Med..

[20] Mason C (2015). The effects of separate and combined dietary weight loss and exercise on fasting ghrelin concentrations in overweight and obese women: a randomized controlled trial. Clin. Endocrinol..

[21] Tymitz K, Engel A, McDonough S, Hendy MP, Kerlakian G (2011). Changes in ghrelin levels following bariatric surgery: review of the literature. Obes. Surg..

[22] Xu HC (2019). Systematic review and meta-analysis of the change in ghrelin levels after Roux-en-Y gastric bypass. Obes. Surg..

[23] Foster-Schubert KE (2005). Human plasma ghrelin levels increase during a one-year exercise program. J. Clin. Endocrinol. Metab..

[24] Au CC, Furness JB, Brown KA (2016). Ghrelin and breast cancer: emerging roles in obesity, estrogen regulation, and cancer. Front. Oncol..

[25] Markowska A, Ziółkowska A, Jaszczyńska-Nowinka K, Madry R, Malendowicz LK (2009). Elevated blood plasma concentrations of active ghrelin and obestatin in benign ovarian neoplasms and ovarian cancers. Eur. J. Gynaecol. Oncol..

[26] Malendowicz W, Ziolkowska A, Szyszka M, Kwias Z (2009). Elevated blood active ghrelin and unaltered total ghrelin and obestatin concentrations in prostate carcinoma. Urologia Internationalis.

[27] Chan DSM (2014). Body mass index and survival in women with breast cancer-systematic literature review and meta-analysis of 82 follow-up studies. Ann. Oncol..

[28] Harrigan M (2016). Randomized trial comparing telephone versus in-person weight loss counseling on body composition and circulating biomarkers in women treated for breast cancer: the lifestyle, exercise, and nutrition (LEAN) study. J. Clin. Oncol..

[29] Suzuki K, Jayasena CN, Bloom SR (2012). Obesity and appetite control. Exp. Diabetes Res.

[30] Havel PJ (2001). Peripheral signals conveying metabolic information to the brain: short-term and long-term regulation of food intake and energy homeostasis. Exp. Biol. Med..

[31] Cummings DE (2001). A preprandial rise in plasma ghrelin levels suggests a role in meal initiation in humans. Diabetes.

[32] Jiralerspong S (2013). Obesity, diabetes, and survival outcomes in a large cohort of early-stage breast cancer patients. Ann. Oncol..

[33] Sanft T (2018). Randomized controlled trial of weight loss versus usual care on telomere length in women with breast cancer: the lifestyle, exercise, and nutrition (LEAN) study. Breast Cancer Res. Treat..

[34] *Dietary Guidelines for Americans*, www.dietaryguidelines.gov.

[35] Rock CL (2012). Nutrition and physical activity guidelines for cancer survivors. CA Cancer J. Clin..

[36] Puklin, L. et al. Metadata record for the article: Randomized Trial of Weight Loss on Circulating Ghrelin Levels Among Breast Cancer Survivors. *figshare*10.6084/m9.figshare.14356184 (2021). 10.1038/s41523-021-00260-6 PMC8113314 33976224

