# Supplementary material for: Randomized trial of weight loss on circulating ghrelin levels among breast cancer survivors
Source: NPJ Breast Cancer. 2021 May 11;7:49. doi: 10.1038/s41523-021-00260-6 (PMC8113314; doi:10.1038/s41523-021-00260-6)
Supplement: Supplementary file 1 — Reporting Summary [file 41523_2021_260_MOESM1_ESM.pdf]

nature research

Corresponding author(s): Leah Puklin, MPH

Last updated by author(s): Mar 18, 2021

Reporting Summary

Nature Research wishes to improve the reproducibility of the work that we publish. This form provides structure for consistency and transparency in reporting. For further information on Nature Research policies, see our Editorial Policies and the Editorial Policy Checklist.

Statistics

For all statistical analyses, confirm that the following items are present in the figure legend, table legend, main text, or Methods section.

n/a	Confirmed	
☐	☒	The exact sample size (*n*) for each experimental group/condition, given as a discrete number and unit of measurement
☐	☒	A statement on whether measurements were taken from distinct samples or whether the same sample was measured repeatedly
☐	☒	The statistical test(s) used AND whether they are one- or two-sided<br>*Only common tests should be described solely by name; describe more complex techniques in the Methods section.*
☐	☒	A description of all covariates tested
☐	☒	A description of any assumptions or corrections, such as tests of normality and adjustment for multiple comparisons
☐	☒	A full description of the statistical parameters including central tendency (e.g. means) or other basic estimates (e.g. regression coefficient) AND variation (e.g. standard deviation) or associated estimates of uncertainty (e.g. confidence intervals)
☐	☒	For null hypothesis testing, the test statistic (e.g. *F*, *t*, *r*) with confidence intervals, effect sizes, degrees of freedom and *P* value noted<br>*Give P values as exact values whenever suitable.*
☒	☐	For Bayesian analysis, information on the choice of priors and Markov chain Monte Carlo settings
☒	☐	For hierarchical and complex designs, identification of the appropriate level for tests and full reporting of outcomes
☐	☒	Estimates of effect sizes (e.g. Cohen's *d*, Pearson's *r*), indicating how they were calculated

Our web collection on statistics for biologists contains articles on many of the points above.

Software and code

Policy information about availability of computer code

Data collection	The codes developed during this study are available upon reasonable request. Analyses were performed using SAS software version 9.4 (Cary, NC).
Data analysis	The codes developed during this study are available upon reasonable request. Analyses were performed using SAS software version 9.4 (Cary, NC).

For manuscripts utilizing custom algorithms or software that are central to the research but not yet described in published literature, software must be made available to editors and reviewers. We strongly encourage code deposition in a community repository (e.g. GitHub). See the Nature Research guidelines for submitting code & software for further information.

Data

Policy information about availability of data

All manuscripts must include a data availability statement. This statement should provide the following information, where applicable:

- Accession codes, unique identifiers, or web links for publicly available datasets
- A list of figures that have associated raw data
- A description of any restrictions on data availability

The data to support the findings of this study are not publicly available in order to protect patient privacy. A complete de-identified patient-level dataset, study protocol, and statistical analysis plan will be made available to researchers upon request until December 2025 by contacting the corresponding author.

Field-specific reporting

Please select the one below that is the best fit for your research. If you are not sure, read the appropriate sections before making your selection.

☐ Life sciences ☒ Behavioural & social sciences ☐ Ecological, evolutionary & environmental sciences

For a reference copy of the document with all sections, see nature.com/documents/nr-reporting-summary-flat.pdf

Behavioural & social sciences study design

All studies must disclose on these points even when the disclosure is negative.

Study description	The Lifestyle Exercise and Nutrition (LEAN) study was a Phase III randomized controlled weight loss trial (NCT02109068 and NCT02110641), registered in January 2011 and November 2013 respectively, evaluating the effectiveness of in-person or telephone-based weight loss counseling versus usual care on changes in body composition, physical activity, diet, and serum biomarkers over 6 months in 151 breast cancer survivors.
Research sample	Breast cancer survivors were recruited between June 1, 2011 and February 1, 2016. Participants were identified through medical oncology clinics or self-referred via study brochures in the Breast Center at Smilow Cancer Hospital at Yale-New Haven Hospital and the Yale Cancer Center Survivorship Clinic.
Sampling strategy	Details surrounding the eligibility criteria, recruitment and study design have been described in previously published literature. Primary results manuscript which has been referenced in the submitted paper. Harrigan, Maura, et al. "Randomized trial comparing telephone versus in-person weight loss counseling on body composition and circulating biomarkers in women treated for breast cancer: the lifestyle, exercise, and nutrition (LEAN) study." Journal of Clinical Oncology 34.7 (2016): 669.
Data collection	Medical record review and questionnaires were used to determine disease stage, surgery, adjuvant therapy, endocrine therapy, self-reported weight, and comorbidities at baseline and 6 months. A fasting (≥8 h) blood draw was performed at baseline and 6 months. Total serum ghrelin levels were measured using a commercial human ghrelin ELISA (enzyme-linked immunosorbent assays) kit (BMS2192, ThermoFisher Scientific, Waltham, MA).
Timing	Breast cancer survivors were recruited between June 1, 2011, and February 1, 2016. The intervention lasted 6 months.
Data exclusions	Of the 151 LEAN participants, 149 had baseline serum ghrelin measurements (91 intervention group, 58 control group). Six-month data were available for 128 women with 14 participants discontinued or lost to follow-up and an additional 7 participants missing follow-up blood draws (76 intervention group, 52 control group)
Non-participation	Over half (60.2%) of participants randomized to intervention attended all 11 weight loss counseling sessions, and 80.6% attended at least 80% of the counseling sessions.
Randomization	The study was a three-arm (1:1:1) randomized trial comparing in-person versus telephone weight loss counseling versus usual care/control on baseline to 6-month changes in body composition, physical activity, diet, and serum biomarkers.

Reporting for specific materials, systems and methods

We require information from authors about some types of materials, experimental systems and methods used in many studies. Here, indicate whether each material, system or method listed is relevant to your study. If you are not sure if a list item applies to your research, read the appropriate section before selecting a response.

Materials & experimental systems

n/a	Involved in the study	
☒	☐	Antibodies
☒	☐	Eukaryotic cell lines
☒	☐	Palaeontology and archaeology
☒	☐	Animals and other organisms
☐	☒	Human research participants
☐	☒	Clinical data
☒	☐	Dual use research of concern

Methods

n/a	Involved in the study	
☒	☐	ChIP-seq
☒	☐	Flow cytometry
☒	☐	MRI-based neuroimaging

Human research participants

Policy information about studies involving human research participants

Population characteristics	Eligible participants were breast cancer survivors with a BMI ≥ 25.0 kg/m2, diagnosed in the 5 years before enrollment with stage 0 to 3 breast cancer, who had completed chemotherapy and/or radiation therapy at least 3 months before enrollment.

	Women had to be physically able to walk, agree to be randomly assigned, and give informed consent to participate in all study activities. They had to be accessible by telephone and English literate. Women were ineligible if they were pregnant or intending to become pregnant in the next year, had experienced a recent (past 6 months) stroke or myocardial infarction, or had severe uncontrolled mental illness.
Recruitment	Breast cancer survivors were recruited between June 1, 2011 and February 1, 2016. Participants were identified through medical oncology clinics or self-referred via study brochures in the Breast Center at Smilow Cancer Hospital at Yale-New Haven Hospital and the Yale Cancer Center Survivorship Clinic.
Ethics oversight	The Yale School of Medicine Human Investigation Committee approved all procedures, including written informed consent.

Note that full information on the approval of the study protocol must also be provided in the manuscript.

Clinical data

Policy information about clinical studies

All manuscripts should comply with the ICMJE guidelines for publication of clinical research and a completed CONSORT checklist must be included with all submissions.

Clinical trial registration	NCT02109068 and NCT02110641
Study protocol	Primary results manuscript which has been referenced in the submitted paper. Harrigan, Maura, et al. "Randomized trial comparing telephone versus in-person weight loss counseling on body composition and circulating biomarkers in women treated for breast cancer: the lifestyle, exercise, and nutrition (LEAN) study." Journal of Clinical Oncology 34.7 (2016): 669.
Data collection	Medical record review and questionnaires were used to determine disease stage, surgery, adjuvant therapy, endocrine therapy, self-reported weight, and comorbidities at baseline and 6 months. A fasting (≥8 h) blood draw was performed at baseline and 6 months. Total serum ghrelin levels were measured using a commercial human ghrelin ELISA (enzyme-linked immunosorbent assays) kit (BMS2192, ThermoFisher Scientific, Waltham, MA).
Outcomes	Primary Outcome: Height (using a stadiometer) and weight were measured at baseline and 6 months. Participants were weighed and measured while wearing light indoor clothing, without shoes. Measurements were rounded up to the nearest 0.1 kg for weight and to the nearest 0.1 cm for height. All measurements, made by the same staff member, were performed and recorded twice in succession, then averaged for analyses.<br>Secondary Outcomes: Measurements were taken at the smallest waist and largest hip circumference areas, rounding up to the nearest 0.1 cm. All measurements, made by the same staff member, were performed and recorded twice in succession, then averaged for analyses.<br><br>Blood draw and serum biomarkers.<br>A fasting (≥8 h) blood draw was performed at baseline and 6 months. All serum samples were stored at -80 degree Celsius until assayed. Total serum ghrelin levels were measured using a commercial human ghrelin ELISA (enzyme-linked immunosorbent assays) kit (BMS2192, ThermoFisher Scientific, Waltham, MA). The serum samples from each individual were analyzed in duplicate, and the absorbance was measured at the wavelength of 450 nm with the reference wavelength of 620 nm for correction using a 96-well BioTek Synergy HT microplate spectrophotometer (BioTek, Winooski, VT). The coefficients of variation for human ghrelin ELISA intra-assay was 1.69% in this study. Serum concentrations of insulin, leptin, and adiponectin were measured using radioimmunoassay kits; IL-6 and TNF-α were measured using high-sensitivity enzyme-linked immunosorbent assay kits; and C-reactive protein (CRP) and glucose were measured using an automated chemistry analyzer. Baseline and 6-month specimens were assayed simultaneously at the end of the study, and participants from all groups were included in each batch of assays. Laboratory technicians were blinded to treatment assignment.<br><br>Dual-energy x-ray absorptiometry scans.<br>Dual-energy x-ray absorptiometry scans were performed to assess body fat, LBM, and BMD at baseline and 6 months with a Hologic 4500 scanner. All scans were evaluated by a Radiologic Technician Certified in Bone Density who was blinded to randomization group.<br><br>Physical activity.<br>At baseline and 6 months, participants completed an interview-administered physical activity questionnaire. The past 6 months of physical activity, including the type, frequency, and duration of 20 activities, were assessed.9<br><br>Pedometers.<br>Yamax pedometers were used to measure number of steps walked per day for 7 days, at baseline and 6 months. Participants recorded the number of steps walked per day from waking until bedtime.<br><br>Dietary intake.<br>Dietary change was assessed by mean group-level changes in daily caloric intake, on the basis of a 120-item food frequency questionnaire, which was developed for the Women's Health Initiative Study.10 Food frequency questionnaires were administered at baseline and 6 months.
